# International Trade and Investment and Food Systems: What We Know, What We Don’t Know, and What We Don’t Know We Don’t Know

**DOI:** 10.34172/ijhpm.2020.202

**Published:** 2020-10-27

**Authors:** Ashley Schram, Belinda Townsend

**Affiliations:** Menzies Centre for Health Governance, School of Regulation and Global Governance, Australian National University, Canberra, ACT, Australia.

**Keywords:** Trade and Investment, Food Systems, Unhealthy Commodities, Corporate Power, Synthetic Foods, Governance

## Abstract

**Background:** Globalised and industrialised food systems contribute to human and planetary health challenges, such as food insecurity, malnutrition, and climate change. International trade and investment can serve as a barrier or enabler to food system transformations that would improve health and environmental outcomes.

**Methods:** This article used health impact assessment (HIA) to analyse what we know, what we don’t know, and what we don’t know we don’t know about the role that trade and investment might play in food system transformations to improve human and planetary health.

**Results:** Evidence exists for the link between trade and investment and the spread of unhealthy food commodities, efforts to impede nutrition labelling, and increased concentration of ultra-processed food and beverage product companies. The role of trade and investment in the reduction of animal sources in human diets is emerging and may include challenging measures that restrict the use of terms like ‘milk’ and ‘burger’ in plant-based alternatives and the promotion of plant-based foods through non-tariff barriers and targeted efforts at regulatory harmonisation. Trade disputes may serve as the forum for battles around state discrepancies in the safety and acceptability of technological innovation in the food supply, as was the case with hormone treated beef between the European Union (EU) and the United States. Corporate social responsibility (CSR) obligations are unambitious but represent welcome progress in balancing public and private interests. Finally, introducing greater policy flexibility, transparency, and participation provides opportunities to shape a modern trade and investment system that can respond to future food system challenges in a timely fashion.

**Conclusion:** Research at the intersection of trade and investment and food systems should address emergent food systems issues, particularly those that intersect health and climate, while policy efforts should be future-proofing the flexibility of the trade and investment system to enable food system design that supports improved human and planetary health outcomes.

## Background

Food systems, in their current globalised and industrialised form, are a key contributor to a number of complex human and planetary health challenges. For example, while 1 in 9 people across the globe went hungry in 2018 – the third year in a row that global hunger prevalence increased^1^ – food systems have also been a driving force in the overconsumption of nutrient-poor calories associated with rising rates of overweight and obesity.^2^ Moreover, the increased productivity of food systems which once brought improved nutrition and development to many, though not all, are now a significant cause of deforestation, groundwater depletion, species and biodiversity loss, and greenhouse gases.^3^ These environmental consequences are already negatively impacting food insecure regions across the globe.^4,5^

One channel by which food systems have become more globalised is international trade and investment. Agreements on the terms of trade and investment between states aim to increase the flow of goods, services, and capital across borders. This goal is achieved in many ways, such as: reducing the taxes applied to foreign imports at the border (ie, tariffs) to increase their ability to compete with domestic products; opening new sectors of a domestic economy to foreign investment, or increasing the percentage of foreign ownership allowable within an industry, such that foreign nationals can own a controlling stake in a domestic company; or through processes of regulatory coherence, whereby standards, rules and procedures are harmonised across countries and greater transparency and participation is enshrined in the domestic policy-making process as a part of international obligations. Unlike many international treaties pertaining to human health or the environment, trade and investment agreements have embedded enforcement procedures and financial penalties for noncompliance through dispute settlement, either between states (state-state dispute settlement) or between private investors and states (investor-state dispute settlement [ISDS]).^6^

 This special issue focuses on the political economy of transforming the food system to improve human and planetary health outcomes. It addresses topics such as the need to reduce the high volume of unhealthy commodities and animal products produced by modern food systems, as well as the need to tackle concentrated corporate power while introducing effective corporate social responsibility (CSR) measures. This contribution will specifically focus on the role of international trade and investment agreements and how they will possibly impede or support the food system transformations identified within this special issue.

 The first section of this article will review what we know, that is, those food transformations identified in the special issue that have been studied with at least some level of breadth to date in the trade and investment and food literature (eg, ultra-processed foods, corporate concentration). The second section will move into food transformations that are known emerging issues in the food system, but where relatively little research has been conducted about the intersection with trade and investment (eg, reducing animal products through initiatives such as lab grown meats, CSR obligations). Lastly, the third section attempts to tackle all the food system transformations we don’t know, we don’t know; that is, necessary transformations in the food system that will appear in the future, but at present are unknowable, and how they will intersect with the power and politics of international trade and investment. The very nature of this final section means we cannot be sure what issues will fall here, and thus the discussion centres on opportunities to future-proof trade and investment policy in such a way that it is flexible enough to support healthy and sustainable food systems going forward.

## Key Messages

**Implications for policy makers**

- Limit tariff reductions on unhealthy agricultural products, such as those used primarily in the production of ultra-processed food and beverage products (eg, high fructose corn syrup).
- Implement comprehensive conflict of interest policies that guide private sector participation in trade and investment negotiations and international policy-making forums (eg, Codex).
- Develop evidence-informed regulatory approaches for synthetic food products, rooted in the precautionary principle.
- Protect policy space for health and food system regulation based on recent progressive approaches to health exceptions in trade and investment agreements.
- Reform domestic governance of trade and investment negotiations to enhance transparency and accessible participation from civil society.

**Implications for public**

The international regime of trade and investment (ie, the flow of goods, services, and capital across national borders) is one avenue that could either support or hinder a healthier and more sustainable food system. This review demonstrates that, at present, trade and investment agreements support global food systems that preference ultra-processed food and beverage products and the concentration of power among a few corporate actors. However, calls for reduced animal products in the global food supply could be aided by trade and investment rules that prevent national policies which discriminate against alternative plant-based proteins at the behest of meat and dairy industries. Shifts to synthetic food products, such as lab grown meat, should be carefully monitored, as intellectual property (IP) rights provided by trade and investment agreements could enhance corporate control over the global food supply. Building more policy flexibility and accountability into trade and investment agreements will help future-proof the system to support healthier and more sustainable food systems.

## Methods

The food system transformations addressed in this review (eg, reduced ultra-processed foods, animal products, and corporate power; improved food labelling; as well as enhanced CSR) were derived from the focal topics of the current special issue. The aim was to locate barriers and enablers to these transformations as shaped by the power and politics of the international trade and investment system. Our analysis was guided by a conceptual framework (see Figure) of the trade–food system–nutrition–climate nexus which outlines both the technical (eg, trade and investment agreement provisions) and political (eg, ideas, interests, and institutions) aspects of trade and investment agreements and the relationship with malnutrition and climate change.^7^

**Figure:**
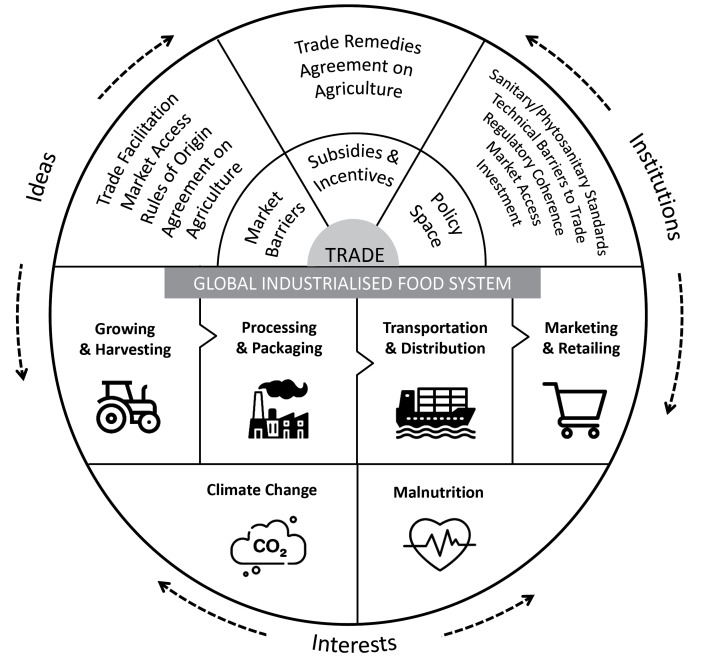
The Trade-Food-System-Nutrition-Climate Nexus (From Friel et al^7^).

We followed the health impact assessment (HIA) process developed by the European Centre for Health Policy which defines HIAs as “a combination of procedures, methods and tools by which a policy, program or project may be judged as to its potential effects on the health of a population, and the distribution of those effects within the population” (p. 4).^8^ This method suggests one of three investigative approaches: (1) a health impact appraisal; (2) a health impact analysis; or (3) a health impact review. Health impact appraisal is a rapid systematic assessment of the policy by experts and stakeholders, primarily based on existing data. Health impact analysis involves new data collection and analysis, while health impact reviews aim to give an overview without necessarily trying to disentangle the precise impact of the various parts of the policy on any specific aspect of health.^8^

The aim of this paper was most closely aligned with a health impact appraisal, such that we were drawing on knowledge from policy area experts to forecast which components of trade and investment agreements would pose barriers or enablers to more healthy and sustainable food systems through the identified food system transformations. The combined expertise of the authors in trade and investment policy, food systems, and public health, was used to scope the areas for assessment in each of the three categories (eg, known, emerging, and unknown intersections between trade and investment and food systems) based on the conceptual framework. A rapid review was conducted for the selected known issues (see Table for search terms). Existing knowledge of the literature and policy spaces was used to forecast emerging issues (eg, intersections between trade and investment and reducing dietary animal sources) and unknown intersections. In this case, unstructured searches of grey literature, media publications, and the Google Scholar database were employed.^9^

**Table:** Search Strategy for Known Trade Tensions in the Focal Areas of This Special Issue

**Known Categories**	**Preliminary Search Terms**	**Databases**
Ultra-processed food and beverage products	Ultra-processed food*, food processing, processing food*, ultra-processed product*, beverage*, sugar sweetened beverage*, SSB	Scopus, ProQuest and Web of Science
Food labelling	Food labelling*, food labeling*, (food AND labelling), (food AND labeling), nutrition labelling*, nutrition labeling*, (nutrition AND labeling), (nutrition AND labelling)	Scopus, ProQuest and Web of Science
Corporate power	Big Food, transnational corporations, food industry, (intellectual property* AND seed), food industry lobbying	Scopus, ProQuest and Web of Science
Trade	Trade, trade agreement*, trade policy, (trade AND regulatory coherence), (trade AND transparency)	Scopus, ProQuest and Web of Science

Abbreviation: SSB, sugar sweetened beverage.

## Results and Discussion

###  Trade and Food Systems: What We Know 

####  Ultra-processed Food and Beverage Products

Trade and investment has been documented as a structural driver for increased production, supply and consumption of unhealthy commodities through a number of pathways.^6,10,11^ Tariff reductions on ultra-processed foods and beverages, for example, have been shown to lead to increased supply and consumption as a result of higher importation and lower prices due to greater competition. The impacts of tariff reductions on increased consumption of processed foods has been documented in North and Central America, South East and Central Asia, Africa, and the Pacific.^10,12-17^ For example, studies have revealed an increase in the supply and consumption of caloric sweeteners, namely high-fructose corn syrup, in Canada following tariff reductions in the North American Free Trade Agreement (NAFTA) between the United States, Canada and Mexico.^18^

Foreign direct investment (FDI) by multinational companies into local production of ultra-processed food and beverages is an even more important channel than trade. Between 1980 and 2000 – a period of extensive trade and investment liberalisation around the world – US FDI into food processing companies grew from US$9 billion to US$36 billion globally. This was accompanied by an increase in sales from US$39.2 billion to US$150 billion. Processed food exports, by comparison, generated only US$30 billion in sales in 2000. Increased FDI has also facilitated the growth of fast-food retail outlets and supermarkets, leading to increased demand for unhealthy products^19^ as well as introducing new unhealthy products into countries and regions.^20^ The association between the liberalisation of investment terms, increased FDI from major multinational food companies into production and retail, and increased sales and consumption of ultra-processed foods has been well-documented in several regions across the globe.^14-16,21-23^

 Acts of regulatory harmonisation may restrict public health efforts to introduce new regulations to control the supply and consumption of unhealthy food products. This is captured more comprehensively in the next section on the intersection of trade and investment and food labelling policy.

The impact of the aforementioned pathways is that trade and investment agreements are shaping the nutrition and food system globally and locally, with implications for population health. A recent systematic review of the quantitative evidence of trade and investment agreements for health concluded that the implementation of agreements is correlated with “higher cardiovascular disease incidence and higher body mass index.”^24^

####  Food Labelling

A growing body of literature demonstrates that front-of-pack labels that are graphical, provide information on nutritional quality, and are placed on the primary display panel can promote consumer nutrition knowledge and healthy food choices.^25-28^ The World Health Organization (WHO) recommends the implementation of front-of-pack nutrition labels as part of a comprehensive approach to promoting healthy diets and improving nutrition.^29^ However, it has been well-documented that many countries have experienced impediments to new labelling regulations through trade queries and challenges using the World Trade Organisation (WTO) Technical Barriers to Trade (TBT) Agreement.

The WTO TBT committee provides a forum for review of technical regulations affecting trade, guided by the principles that regulations should not be discriminatory or unnecessarily trade-restrictive, and should be based on relevant international standards.^30^ Analysis of trade challenges through the TBT committee between 1995 and 2016 identified challenges to 46 food regulatory measures, the most common being labelling regulations.^31^ Ecuador, Chile, Indonesia, Peru and Thailand have faced questioning regarding their nutritional labelling laws with concerns raised by other countries regarding appropriate justification for labelling regulations and whether there is adequate scientific evidence and consistency with international standards.^30^ These pressures have led to regulatory chill with some countries abandoning or weakening their labelling laws. For example, after the United States opposed Thailand’s proposed front of pack ‘traffic light’ labelling system for snack food products, Thailand abandoned the policy and implemented a different labelling system.^11^

####  Corporate Power 

The current trade and investment system has a tendency to preference larger actors. Multinational food corporations have used FDI to consolidate power both horizontally (within a single industry, eg, fast food retail) and vertically (across levels of industry, eg, production, processing, distribution and retail).^32^ This concentration has given a handful of players more power to set the terms of trade through greater influence over states during negotiations.^33^ The sophisticated global supply chains engineered by these companies have also been shown to preference ultra-processed food products given their high profit margins, increased transportability, long shelf lives, and branding capacity.^13,15,16,18,21,34,35^

It was revealed in 2016, that 10 companies control almost every food and beverage brand globally, with a combined revenue of almost $400 billion in 2015.^36^ This rising level of financial capital is then translated into political capital through lobbying and political donations, including during new trade and investment negotiations^33,37^ allowing companies to secure more favourable terms.

One avenue that shifts the balance of power in food production is the disruption of the informal system of saving and sharing seeds vital to the viability of small-scale producers in low-income countries. Under the rules of the WTO, all member states must provide intellectual property (IP) protection for plant varieties through some form of legal system. While many low- and middle-income countries have introduced laws that are WTO consistent, preserving the ability of farmers to save, resow, and exchange seeds^38^; new regional trade and investment agreements have increased the pressure on these countries to deepen their IP regime. For example, the final text of the Comprehensive and Progressive Agreement for Trans Pacific Partnership (CPTPP) includes a commitment by parties to the most recent version of the International Union for the Protection of New Varieties of Plants (known as UPOV 1991). UPOV 1991 requires IP protection for all species of seeds for either 20 or 25 years, and introduces new restrictions on the use, exchange and sale of protected seeds that adversely affects farmers’ livelihoods and food security.^39^ Recently, PepsiCo filed a lawsuit against a small group of Indian farmers for planting and selling PepsiCo’s protected FC5 potato variety for its Lay’s^©^ brand chips without paying royalties, claiming damages of $US142840 for each infringement.^38,40^ After much civil society protest, PepsiCo settled, pending the farmers gave an undertaking to henceforth purchase this specific variety of seeds from the company and sell the potatoes to PepsiCo. As India is not currently a member of UPOV, it is unclear how the dispute would have proceeded with the additional protections and enforcement measures that UPOV provides for corporations like PepsiCo.

Another avenue by which corporate power is enhanced is through rules about transparency and regulatory coherence introduced by trade and investment agreements. These types of provisions offer new opportunities for powerful food and beverage industries to review and provide feedback on government proposed policies and regulations.^10,41^ For example, the final text of the CPTPP contains a provision that “Each Party shall allow persons of the other Parties to participate in the development of technical regulations, standards and conformity assessment procedures by its central government bodies…on terms no less favourable than those it accords to its own persons” (art.8.7, ¶1). New channels enshrining the rights of food and beverage corporations to participate in the development of regulations and policies across CPTPP member countries has raised serious health concerns of regulatory capture or a weakening of regulations and policies to improve the food system.^41^ Granting greater rights to corporations also appears inconsistent with efforts in the public health community to protect policy-making and implementation from vested interests, such as those through the WHO’s action plan for the prevention and control of non-communicable diseases or its framework of engagement with non-state actors.^42^

Greater representation of industry interests in the policy process may deter or weaken nutrition-related health regulations. For example, during renegotiations of NAFTA, US food industry groups were pushing for provisions to limit the ability to require consumer warnings on the front of sugary drinks and high fat processed foods.^43^ They were also lobbying for new provisions to prevent “unjustified” trade restrictions including TBT measures and restrictions on marketing, promotion, branding and quantity of food.^44^ Analysis of the final text of the agreement finds tighter restrictions on new regulatory measures,^45^ although time is needed to see how these changes will be manifested and the impact they will produce. It has been documented in the literature that trade and investment agreements generally preference a global and industrialised food system controlled by a few major players, which in turn provides these actors with greater influence over the future terms of trade, allowing them to secure even more control over the food system. This influence has been used to prevent government regulations designed to deter consumers from ultra-processed food and beverage products (eg, labelling initiatives) and the enhancement of monopoly rights (eg, seeds). In order for trade and investment agreements to better support healthy and sustainable food system transformations, policy-makers should consider actions such as limiting tariff reductions on unhealthy agricultural products, including those used primarily in the production of ultra-processed food and beverage products (eg, high fructose corn syrup). Policy-makers should also consider implementing comprehensive conflict of interest policies that guide private sector participation in trade and investment negotiations.

###  Trade and Food Systems: What We Don’t Know 

####  Plant-Based and Synthetic “Meat” and “Dairy”

One of the recommendations that emerged from both the Lancet Global Syndemic Commission^46^ and the EAT-Lancet Commission^47^ was the need to reduce animal source foods, such as red meat – as nutritionally-appropriate within local contexts – for a healthy and sustainable diet. There are multiple ways in which such a reduction could be achieved. One avenue is to replace animal protein products with naturally occurring plant-based proteins, such as beans, legumes, nuts, grains, and the like. Trade and investment is unlikely to present any barriers to this avenue. In fact, by lowering tariffs and harmonising non-tariff barriers (eg, food standards) related to these goods, such agreements could actually facilitate this pathway to meat reduction.

Another way in which people reduce their consumption of animal source foods is by substituting their diet with some form of processed meat or dairy alternatives, such as veggie ‘burgers’ or soy ‘milk.’ An increase in consumer demand and sales of these products is required in order to see a significant supply side change within food systems. Marketing and advertising is an important avenue for building consumer acceptability and desirability for new food products.^48,49^

One of the most contentious issues in the marketing and advertising of meat and dairy alternatives has been the ability of such products to use meat or dairy monikers, even when preceded by the term “plant-based.” This is still largely a battle between the meat and dairy industries on the one hand and the alternative protein-based food industry on the other, playing out primarily at the domestic level. In Canada, the matter is settled on the dairy front (no doubt due to its highly influential dairy farmers lobby): alternative non-dairy beverages cannot be labelled ‘milk.’^50^ However, the battle continues in countries such as the US and Australia.^51,52^ The question might be posed though, could countries like Canada be vulnerable to a trade challenge for implementing such restrictions?

The Codex Alimentarius provides the officially recognised standards, codes of practice, guidelines, and other recommendations relating to foods, food production, and food safety within the trade and investment system. Codex defines milk as “the normal mammary secretion of milking animals”^53^ and meat as the edible part of “of any mammal slaughtered in an abattoir.”^54^ In its dairy standards, Codex further stipulates that only a food complying with this definition may be named ‘milk.’ However, a later provision introduces ambiguities by noting that the previous provision “shall not apply to the name of a product the exact nature of which is clear from traditional usage or when the name is clearly used to describe a characteristic quality of the non-milk product.”

This may leave quite a bit of space for interpretation within countries, some of which have been reluctant to enforce such distinctions. For example, in the United States several federal courts have rejected the argument that calling non-cow milk ‘milk’ is misleading. One ruling from a Ninth Circuit court in California noted that “it is simply implausible that a reasonable consumer would mistake a product like soymilk or almond milk with dairy milk from a cow…the first words in the products’ names should be obvious enough to even the least discerning of consumers.”^55^ Similarly, in December 2019, a US federal court ruled that restricting the use of terms of traditional meat products was a violation of free speech, and that plant-based company Tofurky could continue to call its products ‘plant-based sausage.’ In the ruling the judge noted that “the State appears to believe that the simple use of the word ‘burger,’ ‘ham,’ or ‘sausage’ leaves the typical consumer confused, but such a position requires the assumption that a reasonable consumer will disregard all other words found on the label” and that such an assumption was unwarranted.^52^

As noted above, nutrition labelling has been challenged based on consistency with international standards, as well as a range of other rationales.^30^ Findings such as the ones above from US federal courts indicate that an argument could be put forth by alternative protein-based food industries (such as producers of alt-milk products) that measures such as those taken in Canada limiting the term ‘milk’ (likely on behest of the dairy industry) are more trade restrictive than necessary, thus limiting their ability to market and sell their products. As plant-based replacements take a larger share of the market, it would not be impossible to imagine a trade challenge being raised, if only informally through the TBT committee.

Synthetic, cultured, or lab-grown ‘meat’ will likely face similar battles over the use of meat monikers.^56^ However, its reliance on novel laboratory technologies which have accrued relatively little scientific evidence in terms of safety will also necessitate new scientific risk assessments. The question in terms of trade and investment will be, to what extent the increased harmonisation of standards – and in some treaties the requirement to accept another country’s standards as equivalent – reduces policy space for domestic decision-making about the safety and acceptability of cultured meat in the food supply. Such a question inevitably calls to mind one of the most intractable and acrimonious trade disputes in the history of the WTO: the US-European Union (EU) beef hormone dispute. The essence of the dispute was the EU’s ban on the importation of meat that contained artificial beef growth hormones approved for use in the US but not approved in the EU – at the heart of which was a disagreement on the outcomes of scientific risk assessments and the role of the precautionary principle in protecting public safety.^57^ Similar cases could very easily emerge around cultured meat.

In addition to potentially competing notions of safety and consumer acceptability, careful consideration should be given to the capacity for technologically produced food products to enhance corporate concentration in the food system. Trade and investment agreements provide expansive and enforceable IP protections through patent rights, thus lab-grown varieties of meat and dairy open new avenues for multinationals to expand their ownership and control of the global food supply. Likewise, the impacts on already imbalanced agricultural relationships between those countries with the capacity to compete in such ‘food tech’ markets and those without^58^ should be thoughtfully deliberated while there is still time. It is also worth noting that plant-based alternatives and synthetic food products, while reducing animal products in the global diet and supporting environmental aims, may undermine nutritional aims to reduce ultra-processed food products and improve the healthfulness of the food supply.^59^

####  Corporate Social Responsibility Obligations

Calls for CSR in the food sector are echoed within the international investment system.^60^ While trade and investment are frequently grouped together, these systems functioned separately until the mid-nineties when NAFTA incorporated an investment treaty as a chapter within the agreement, launching a much greater integration of these two regimes. Despite these connections, trade and investment still operate from quite distinct infrastructure and are frequently negotiated independently.

Historically, investment agreements have been designed to bestow greater rights upon corporations and greater obligations upon states in the name of promotion and protection of FDI, initially into developing regions. For investors from developed states the system was put in place to ensure fair and effective procedures in the event that a dispute arose – such as the expropriation of foreign investments by governments in socialist and newly developing states. Developing states were encouraged to sign up to these obligations in order to drive inward FDI, thereby creating an influx of capital, knowledge and technology transfer, as well as new employment opportunities, and increased competition and efficiency in the host state.^61^ However, investment treaties have come under increased public scrutiny, in large part due to ISDS, a system built into investment treaties which allows foreign investors access to private international tribunals to bring forward claims against states for the violation of investor rights for financial compensation. To date, foreign investors have used the system to challenge a wide array of public policy measures, including measures on taxation, chemical and mining bans, environmental restrictions, transportation and disposal of hazardous waste, health insurance, tobacco, the price and delivery of water, and regulations to improve the economic situation of minority populations.^62^

These challenges have led to a legitimacy crisis around investment treaties which has thrust the system into reform, opening up space for progressive ideas. One of these areas has been the piloting of CSR provisions, designed to limit negative social and environmental externalities caused by the activities of multinational companies. At present, 30 investment treaties have introduced CSR provisions, although most remain quite weak at this stage. Canada, for example, in its treaties has begun encouraging a form of self-regulation. Language in the Canada–EU agreement states that the countries agree on “encouraging the development and use of voluntary best practices of CSR by enterprises, such as those in the Organisation for Economic Co-operation and Development (OECD) Guidelines for Multinational Enterprises, to strengthen coherence between economic, social and environmental objectives.” The OECD guidelines cover a range of issues such as human rights, labour rights, and the environment. Likewise, attempts to introduce obligations governing multinational activity use conditional language like ‘should’ or ‘shall,’ as in an agreement between Brazil and Malawi which states that “[t]he investors and their investments shall develop their best efforts to comply with the following voluntary principles and standards for a responsible business conduct and consistent with the laws adopted by the Host Party receiving the investment.”^63^ While CSR obligations within investment treaties are far from optimal, they could go some way to correcting the imbalance between foreign investors and states produced by the treaties themselves, as well as convert ‘softer’ CSR principles into enforceable international obligations.

 Reducing the volume of animal products in the global food supply is an emerging issue. While trade and investment liberalisation has contributed to the increased production and sale of animal products, it equally has the capacity to support an increase in alternative plant-based products. Specifically, it may prevent national policies that restrict the marketing and advertising of plant-based products (eg, soy milk and Tofurky) or the sale of lab-grown meats. Implementing comprehensive conflict of interest policies that guide private sector participation in international policy-making forums (eg, Codex) may be advisable, as well as developing evidence-informed regulatory approaches for synthetic food products, rooted in the precautionary principle. It will be important to consider unintended consequences of food system transformations, such as the capacity for greater IP protections for synthetic foods to increase corporate control over the global food supply or further entrench imbalances between countries.

###  Trade and Food Systems: What We Don’t Know, We Don’t Know

 The challenges belonging in this section are by their very nature unknowable; however, as is oft quoted, the only constant in life is change. We can be assured that new issues will emerge within the food system, and that many of them will inevitably intersect with the trade and investment sector. Consequently, this section discusses opportunities to make a more adaptable and flexible trade and investment system that would more easily enable domestic and international action to address such challenges.

####  Policy Flexibility

One of the clearest needs is enhanced policy flexibility. Trade and investment agreements need to explicitly recognise the dynamic and evolving nature of domestic regulatory systems and not impede action in this space. At present, the most cited protection for policy space in trade agreements is known as the general exception, first employed in the WTO General Agreement on Tariffs and Trade (GATT). This provision permits members to adopt measures that violate GATT if it is ‘ *necessary to protect *human health, animal or plant life or health’ (Article XX[b]) and provided they do not constitute a means of arbitrary or unjustifiable discrimination between countries, or a disguised restriction on trade. This same provision has been copied into countless trade and investment agreements. However, the utility of this provision has been called into question as only one of 44 attempts to invoke this general exception has been successful. In the 33 cases where the exception was deemed to be relevant, the majority (N = 18) failed to establish that measures were ‘necessary to’ protect health.^64^ This provision has seen substantial reform within the investment system. For example a 2018 agreement between Peru and Australia included a provision in the investment chapter, stating that “No claim may be brought under this Section [ISDS] in relation to a measure that is *designed and implemented to* protect or promote public health.” While it remains to be seen whether this provision will produce significantly different outcomes, or whether additional agreements will adopt this revised language, arguably it should lower the burden of proof on states when justifying new policy measures.

There are also opportunities to design more robust food policy in the domestic sphere. For example, one of the most prominent domestic responses to the impacts of trade and investment on nutrition to date, has been the suite of policy measures designed to reduce supply and consumption of imported high-fat meats. The variety of policy measures that have been implemented have met with varied success.^20^ Countries in the Pacific, such as Fiji, Samoa, and Tonga have all implemented or proposed measures tackling specific meat imports (eg, mutton flaps, turkey tails) using a variety of sales bans, import bans, and import quotas. Ghana, on the other hand, took the broader approach of introducing limits on maximum allowable percentages of fat in a variety of meats. Evidence seems to indicate that the measures in Ghana have several potential benefits over the responses in the Pacific, as they are less likely to result in substitution (one high fat meat product for another), will apply to new high-fat meat products introduced in the future, and may be more defensible from a trade and investment perspective (eg, the measure does not discriminate between products or country of origin)^65^ These types of adaptable language and policy both in the trade and investment system, and domestic food policy systems, will assist in tackling future, unknowable issues.

####  Transparency and Participation 

Lack of transparency in trade and investment negotiations means we often don’t know if there are provisions or rules that could create barriers for promoting a healthy and sustainable food system until after agreements are signed. Many governments do not provide consultation processes or information about the issues and provisions under negotiation in new trade agreements. For those that do, public health experts and civil society have expressed strong dissatisfaction for a lack of meaningful consultation and input, and have reported reliance on leaked text to survey what issues might be on the table.^37,66^ This frustration is also evident amongst government health officials in some countries. In Malaysia and Australia, for example, health officials have reported power imbalances making them reliant on trade officials to identify whether a trade agreement might affect health.^66^ In contrast, the United States has domestic industry committees which enable up to 600 industry actors, and a small number of approved non-governmental organizations, to confidentially view sections of negotiation text.^67^

In addition to these power imbalances between market and health actors, there are very few examples of governments implementing robust HIAs of proposed trade treaty text. Thailand has historically been a notable exception with an interdepartmental International Trade and Health Programme to generate evidence-based analyses and requirements in the Constitution for parliamentary approval of trade negotiation frameworks.^68^ However, in the last 5 years these constitutional protections have been removed and there has been a concerted push for Thailand to join the CPTPP despite concerning government health analyses^69^ (at the time of publication Thailand had not yet joined the CPTPP).

Globally, there is also a lack of transparency on who sits on WTO committees, such as the Committee on Sanitary and Phytosanitary Measures which provides a forum for consultations about food safety measures which affect trade. Likewise, the Codex Alimentarius Commission, which informs international food standards employed in the trade and investment system, has been disproportionately influenced by private economic actors, with 662 representatives from industry and only 26 from public interest groups,^70^ a trend which has continued over time.^71^ Overall, there is urgent need for a transformation of national, regional and global trade and investment processes to be more transparent and accountable, and for greater impact assessment and health sector engagement in the process from the beginning.

## Conclusion

 This contribution addressed some of the ways in which international trade and investment agreements might support or impede the types of food system transformations being discussed in this special issue. It highlighted issues that have been well-studied in the trade and food space as well as emerging issues in the food system that we don’t know much about in terms of their intersections with trade and investment. Moreover, it addressed the things we don’t know, we don’t know will become challenges by exploring opportunities to create responsive trade and investment policy to enhance human and planetary health outcomes from food systems. This article was limited in that it only covered issues pertaining to trade and investment that were focal topics in this special issue, rather than a full survey of issues. Additionally, while the coverage of known issues followed a relatively systematic search of the literature, coverage of emerging and unknown issues was reliant on the policy area knowledge of the authors and thus is not reproducible.

 Considerable evidence exists for the link between trade and investment and the spread of unhealthy food commodities, attempted efforts to impede nutrition labelling using trade mechanisms, as well as increased concentration and influence of companies producing ultra-processed food and beverage products. How trade and investment will support or impede efforts to reduce the contribution of animal sources in human diets is less clear. We suggest here that the trade system could be used to challenge measures that restrict the use of terms like ‘milk’ ‘burger’ or ‘sausage’ in plant-based alternatives on the grounds that they are unnecessary barriers to trade. Assuming that such restrictions limit the marketing of these products, thus reducing consumer recognition and acceptability and sales, the trade system could actually facilitate a reduction in animal food products by removing marketing restrictions of plant-based alternative products. Plant-based foods and protein sources could also be promoted through non-tariff barriers, such as targeted efforts around trade facilitation and regulatory harmonisation. Emerging issues around meat cultured in labs calls forth previous protracted battles at the WTO around hormone treated beef between the EU and the United States and may once again ignite trade wars rooted in opposing views on safety and consumer acceptability. It is too early to say what effect CSR obligations in investment treaties will have, and while one could hope that they will become more ambitious over time, they represent welcome progress in balancing public and private interests in these deals. Finally, it explored the opportunities to mitigate future challenges—what we don’t know we don’t know—by creating trade and investment policy that is responsive to changing needs, in order to enhance human and planetary health outcomes from the food system.

## Ethical issues

 The method of this study was to analyse the secondary data. Therefore, ethical approval is not required.

## Competing interests

 Authors declare that they have no competing interests.

## Authors’ contributions

 AS and BT contributed to the conceptualisation, analysis, and writing of the manuscript.
